# ARTD10 substrate identification on protein microarrays: regulation of GSK3β by mono-ADP-ribosylation

**DOI:** 10.1186/1478-811X-11-5

**Published:** 2013-01-19

**Authors:** Karla LH Feijs, Henning Kleine, Anne Braczynski, Alexandra H Forst, Nicolas Herzog, Patricia Verheugd, Ulrike Linzen, Elisabeth Kremmer, Bernhard Lüscher

**Affiliations:** 1 Institute of Biochemistry and Molecular Biology, Medical School, RWTH Aachen University, Pauwelsstraße 30, 52074, Aachen, Germany; 2 Helmholtz Zentrum München, Institute for Molecular Immunology, Marchioninistr. 25, 81377, München, Germany; 3 Present addresses: Abbott GmbH & Co. KG, Max-Planck-Ring 2a, 65205, Wiesbaden, Germany (HK; 4 Department of Neurology, Medical School, RWTH Aachen University, 52074, Aachen, Germany (AB; 5 Home office, Schoppershofstraße 65, 90489, Nürnberg, Germany (AB

**Keywords:** ARTD, Posttranslational modification, ADP-ribosyltransferase, PARP10, PARP14, ARTD8, NAD^+^, Kinase activity, Regulation

## Abstract

**Background:**

Although ADP-ribosylation has been described five decades ago, only recently a distinction has been made between eukaryotic intracellular poly- and mono-ADP-ribosylating enzymes. Poly-ADP-ribosylation by ARTD1 (formerly PARP1) is best known for its role in DNA damage repair. Other polymer forming enzymes are ARTD2 (formerly PARP2), ARTD3 (formerly PARP3) and ARTD5/6 (formerly Tankyrase 1/2), the latter being involved in Wnt signaling and regulation of 3BP2. Thus several different functions of poly-ADP-ribosylation have been well described whereas intracellular mono-ADP-ribosylation is currently largely undefined. It is for example not known which proteins function as substrate for the different mono-ARTDs. This is partially due to lack of suitable reagents to study mono-ADP-ribosylation, which limits the current understanding of this post-translational modification.

**Results:**

We have optimized a novel screening method employing protein microarrays, ProtoArrays®, applied here for the identification of substrates of ARTD10 (formerly PARP10) and ARTD8 (formerly PARP14). The results of this substrate screen were validated using *in vitro* ADP-ribosylation assays with recombinant proteins. Further analysis of the novel ARTD10 substrate GSK3β revealed mono-ADP-ribosylation as a regulatory mechanism of kinase activity by non-competitive inhibition *in vitro*. Additionally, manipulation of the ARTD10 levels in cells accordingly influenced GSK3β activity. Together these data provide the first evidence for a role of endogenous mono-ADP-ribosylation in intracellular signaling.

**Conclusions:**

Our findings indicate that substrates of ADP-ribosyltransferases can be identified using protein microarrays. The discovered substrates of ARTD10 and ARTD8 provide the first sets of proteins that are modified by mono-ADP-ribosyltransferases *in vitro*. By studying one of the ARTD10 substrates more closely, the kinase GSK3β, we identified mono-ADP-ribosylation as a negative regulator of kinase activity.

## Lay abstract

Newly synthesized proteins need (i) to find their way within a cellular environment and (ii) to be controlled in their functionality. Small modifications termed post-translational modifications, are added to these proteins to guide them to different locations, regulate their activity or induce their breakdown. ADP-ribosylation is a post-translational modification performed by enzymes that use the cofactor NAD^+^ to add ADP-ribose to a substrate protein. Some enzymes, like ARTD10, transfer only one ADP-ribose unit, whereas others, such as ARTD1, transfer multiple units to form chains of ADP-ribose. It is currently not known which proteins can be mono-ADP-ribosylated or what the consequence thereof is. In this study, we screened approximately 8000 proteins on microarrays to test which proteins can be modified by ARTD10 and ARTD8. Subsequently, we validated some of the identified proteins in *in vitro* enzymatic assays and could confirm that ARTD10 and ARTD8 transfer ADP-ribose to these proteins. Next, we investigated what the consequence of mono-ADP-ribosylation is for the ARTD10 substrate GSK3β, a kinase that controls many physiological processes. We found that mono-ADP-ribosylated GSK3β is less active than the non-modified protein. Finally, we expressed ARTD10 and GSK3β together in cells and measured lower GSK3β activity in the presence of ARTD10. In summary this study provides the first substrates of the mono-ADP-ribosyltransferases ARTD10 and ARTD8. Moreover, we could show that mono-ADP-ribosylation inhibits the activity of a target protein, *in vitro* and in cells. These first investigations of a mono-ADP-ribosylated protein show that this modification might have important roles in signaling processes.

## Background

ADP-ribosylation is a posttranslational modification where ADP-ribose is transferred from the co-factor β-NAD^+^ onto a substrate, catalyzed by ADP-ribosyltransferases (ARTs). The eukaryotic transferases can be divided into two groups, the extracellular ARTCs (formerly ARTs) and the intracellular ARTDs (formerly PARPs) [1]. The C and D refer to C2/C3 and diphtheria toxin-like ARTs, respectively, which represent the two distinct structures of catalytic domains that can be distinguished [1]. Of the ARTD family with 18 members [2], only class 1 enzymes are capable of forming polymers of ADP-ribose (PAR). Class 2 enzymes lack the catalytic glutamate necessary to support the transition state during the enzymatic reaction. Instead, they use substrate-assisted catalysis to transfer a single ADP-ribose unit onto substrates [3]. During this process the activating glutamate of the substrate is subsequently ADP-ribosylated, consequently the modified glutamate is not available for a following second catalytic step and thus the reaction is limited to mono-ADP-ribosylation. Class 3 members are proposed to be inactive due to the inability to bind β-NAD^+^[3].

Poly-ADP-ribosylation by ARTD1 (formerly PARP1) has been investigated most thoroughly and is best known for its role in DNA damage repair and the control of chromatin and gene transcription [4-6]. In addition to ARTD1, ARTD2 (formerly PARP2) also participates in DNA repair and *Artd1/Artd2* double knockout animals do not survive [7,8]. ARTD5/6 (formerly Tankyrase 1/2) play a role in Wnt signaling [9-11] and in controlling the stability of the adaptor 3BP2, mutations of which are mechanistically linked to Cherubism [12,13]. The poly-ADP-ribose chains do not directly regulate the molecules they are synthesized on, but for example indirectly reduce ARTD1 activity by disturbing the interaction of ARTD1 with DNA [14] or serve as scaffolds to recruit other proteins through domains such as the WWE domain and macrodomains [4,6,15]. These are often found in DNA repair proteins, explaining the role of ARTD1 in this process [16-19]. Moreover the E3 ubiquitin ligase Iduna (RNF146) interacts with PAR through its WWE domain, providing evidence for poly-ADP-ribosylation indirectly regulating protein stability [9,11,20,21].

In comparison to the polymer forming ARTDs, the mono-ARTDs remain much less well understood, mainly because they have only recently been recognized [3] and because basic research tools such as antibodies recognizing mono-ADP-ribosylated residues are lacking [22]. Some of the mono-ARTDs are suggested to play a role in immunity [23], however most of the data on the mono-ARTDs are mainly descriptive and have not yet revealed the mechanisms underlying the observed phenomena. The founding member of the mono-ARTDs, ARTD10, was identified as interaction partner of MYC [24] and was later on demonstrated to mono-ADP-ribosylate itself and core histones [3]. Although ARTD10 can shuttle into the nucleus, its predominant localization is cytoplasmic, where it associates at least in part with dynamic p62/SQSTM1-positive bodies [25]. Moreover, ARTD10 seems vital for proliferation, as both knockdown and overexpression inhibit normal cell growth, the latter being dependent on catalytic activity [3,26]. However, it is currently unknown which proteins are modified by ARTD10 that could mediate this growth inhibitory phenotype.

To increase our understanding of mono-ADP-ribosylation, we screened for substrates of the mono-ADP-ribosyltransferases ARTD10 and ARTD8. For this we established the use of protein arrays and were able to identify potential substrates for both enzymes, with a remarkable enrichment of kinases. We focused our further studies on the ARTD10 substrate glycogen synthase kinase 3β (GSK3β), a kinase with broad physiological relevance [27,28], and not only confirmed its mono-ADP-ribosylation, but also found that this modification inhibits enzymatic activity. Moreover, siRNA mediated knockdown of ARTD10 led to an increase in GSK3β activity, suggesting that endogenous mono-ADP-ribosylation occurs. The data reported here give a first insight into the functional relevance of mono-ADP-ribosylation and demonstrate that mono-ADP-ribose can directly regulate the enzymatic activity of one of its acceptor proteins.

## Results

### **ARTD10 and ARTD8 substrate identification using ProtoArrays**

**Figure 1:**
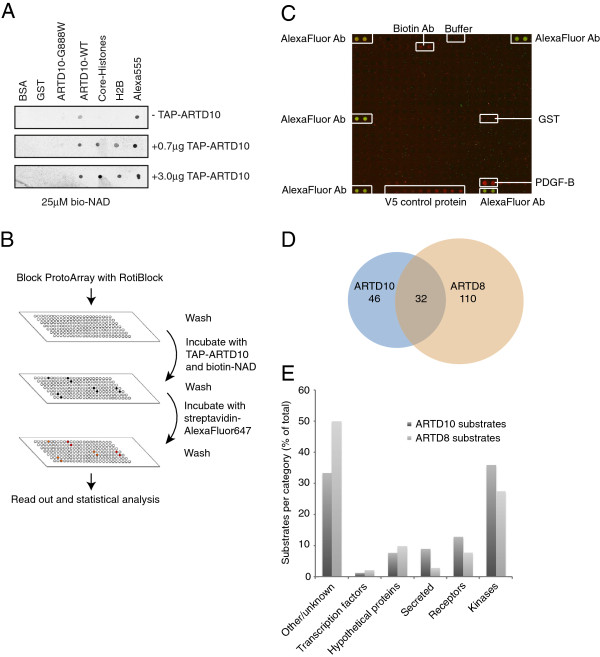
**Application of ProtoArrays® to identify ARTD10 and ARTD8 substrates. A**. Indicated proteins were spotted on nitrocellulose membranes and subsequently incubated with TAP-ARTD10 and biotin-labeled NAD^+^ to optimize array conditions. The signals were detected with HRP-coupled streptavidin. **B.** Schematic representation of ProtoArray®-based screens. The arrays were incubated with ARTD10, ARTD8 or BSA and 25 μM biotin-β-NAD^+^ and visualized using streptavidin-AlexaFluor® 647. **C.** An exemplary magnification of a sub-array from an array incubated with TAP-ARTD10 is shown with controls and top-outlier PDGF-B indicated. **D.** Venn diagram showing the overlap between the identified ARTD8 and ARTD10 substrates. **E.** All putative ARTD8 and ARTD10 substrates sorted according to function.

To gain insight into the molecular function of mono-ADP-ribosylation, we screened for substrates of the mono-ARTDs ARTD10 and ARTD8. Since currently no approaches have been published to carry out a broad screening approach for mono-ADP-ribosyltransferases, we optimized a novel method based on protein microarrays. We performed initial tests to address whether ARTD10 can use biotin-labeled NAD^+^ as co-factor (Additional file 1: Figure S1A). Next we established optimal conditions to enzymatically modify proteins on nitrocellulose-coated slides. ARTD10 and core histones were loaded as positive and BSA and GST as negative controls. These slides were subsequently incubated with tandem affinity purification (TAP)-ARTD10 and biotin-NAD^+^ and were evaluated by horseradish peroxidase (HRP)-coupled streptavidin (Figure 1A). Because TAP-ARTD10 was able to modify immobilized core histones, the only known substrate of ARTD10 besides its ability to automodify [3], on these control slides, we used similar conditions for large-scale screening of ARTD10 and ARTD8 substrates. These screens were performed on protein microarrays, ProtoArrays®, containing approximately 8000 proteins spotted in tandem on a single array. These arrays have been used before to study for example phosphorylation [29]. We incubated the ProtoArrays® with tandem-affinity purified (TAP)-ARTD10, baculo-derived ARTD8 or BSA and biotin-labeled NAD^+^. The modification of proteins on the array was visualized using streptavidin-AlexaFluor® 647 (Figure 1B). A sub-array is shown with different controls and platelet-derived growth factor subunit B (PDGF-B) as top positive signal of ARTD10 (Figure 1C). Moreover a comparison of several ARTD10 and ARTD8 sub-arrays is displayed (Additional file 1: Figure S1B). Proteins that bound biotin or NAD^+^ and NAD^+^-consuming enzymes were identified on the control slides incubated with BSA and biotin-labeled NAD^+^. These signals were manually removed from the positive hits obtained after incubation with ARTD10 or ARTD8 before further analysis, such as the probably automodifying ARTD7 (formerly PARP15). After these adaptations, 78 and 142 proteins were identified as substrates of ARTD10 and ARTD8, respectively, with an overlap of 32 proteins (Figure 1D and Additional file 2: Table S1 and Additional file 3: Table S2). Further analysis revealed that the largest subgroup of ARTD10 substrates was formed by kinases, followed by receptors and growth factors (Figure 1E), kinases also form the largest substrate subgroup of ARTD8 substrates (Figure 1E). This is also reflected by gene ontology (GO) enrichment analysis, where multiple GO terms were found that are related to phosphorylation such as “protein kinase cascade” (GO:0007243) (Additional file 4: Table S3). The total number of substrates identified was quite small, roughly 1% and 1.7% for ARTD10 and ARTD8, respectively, implying that these enzymes are rather selective.

**Figure 2:**
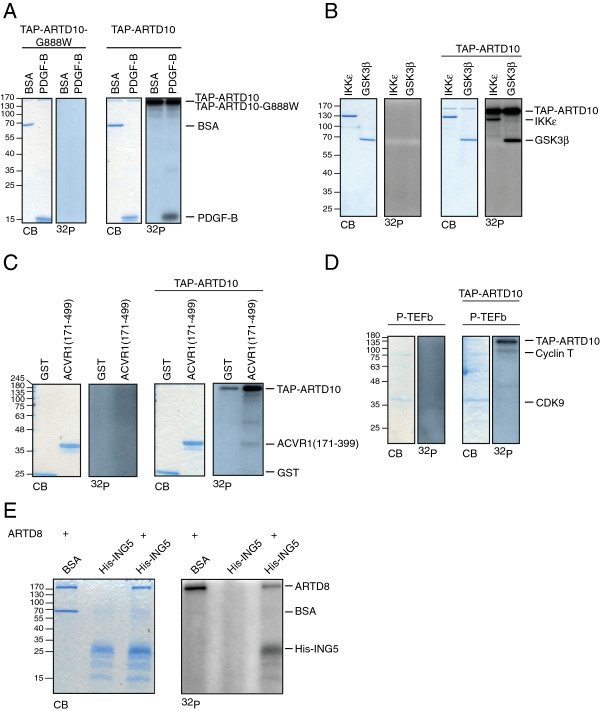
**Validation of the ProtoArray® results on diverse recombinant proteins.** ADP-ribosylation assays with [^32^P]-NAD^+^ as cofactor, analyzed by SDS-PAGE, coomassie blue (CB) staining and autoradiography with **A.** TAP-ARTD10-G888W or TAP-ARTD10 and BSA or PDGF-B **B.** TAP-ARTD10 and IKKε or GSK3 β, or the negative control without ARTD10 **C.** TAP-ARTD10 and ACVR1(171-499), or the negative control without ARTD10 **D.** TAP-ARTD10 and P-TEFb, or the negative control without ARTD10 **E.** His-ARTD8 and GST-ING5, or the negative control without His-ARTD8.

To confirm that the hits identified on the arrays are substrates of ARTD10 and ARTD8, we verified several recombinant proteins in *in vitro* ADP-ribosylation assays. We validated substrates from different subclasses, which were purified with diverse methods to exclude artifacts caused by tags or treatments. The top outlier PDGF-B was verified as ARTD10 substrate in an ADP-ribosylation assay with BSA as negative control (Figure 2A). Interestingly, PDGF-B was identified previously as a factor that can be ADP-ribosylated by an extracellular ADP-ribosyltransferase [30]. Several kinases could also be validated in independent assays, such as NF-κB inhibitor epsilon (IKKε) and GSK3β (Figure 2B). Of the receptor subclass, the activin receptor 1 (ACVR1) was confirmed using the ARTD10 catalytic domain, GST-ARTD10(818-1025), in the ADP-ribosylation assay (Figure 2C). The cyclin T subunit of positive transcription elongation factor B (P-TEFb), could also be modified by the full-length TAP-ARTD10 (Figure 2D).

Inhibitor of growth protein 5 (ING5) was confirmed as ARTD8 substrate (Figure 2E). ARTD8 protects B-cells from apoptosis by modulating key regulators of apoptosis, such as caspase-3 [31] and is also necessary for pro-survival signaling and regulation of glycolytic rate in B-cells [32]. Several substrates identified on the ProtoArrays® are involved in insulin signaling, such as insulin receptor substrate 1 (IRS1) and protein kinase C zeta type (PRKCZ), through which glycolysis can be influenced. Proto-oncogene tyrosine-protein kinase receptor Ret (RET) and protein kinase C iota type (PRKCI) are two of the identified substrates that potentially link ARTD8 with apoptosis. The underlying mechanisms through which ARTD8 mediates its described functions might thus be uncovered through the substrates we discovered. In the following, we concentrated our further studies on one ARTD10 substrate.

### Mono-ADP-ribosylation inhibits GSK3β *in vitro*

**Figure 3:**
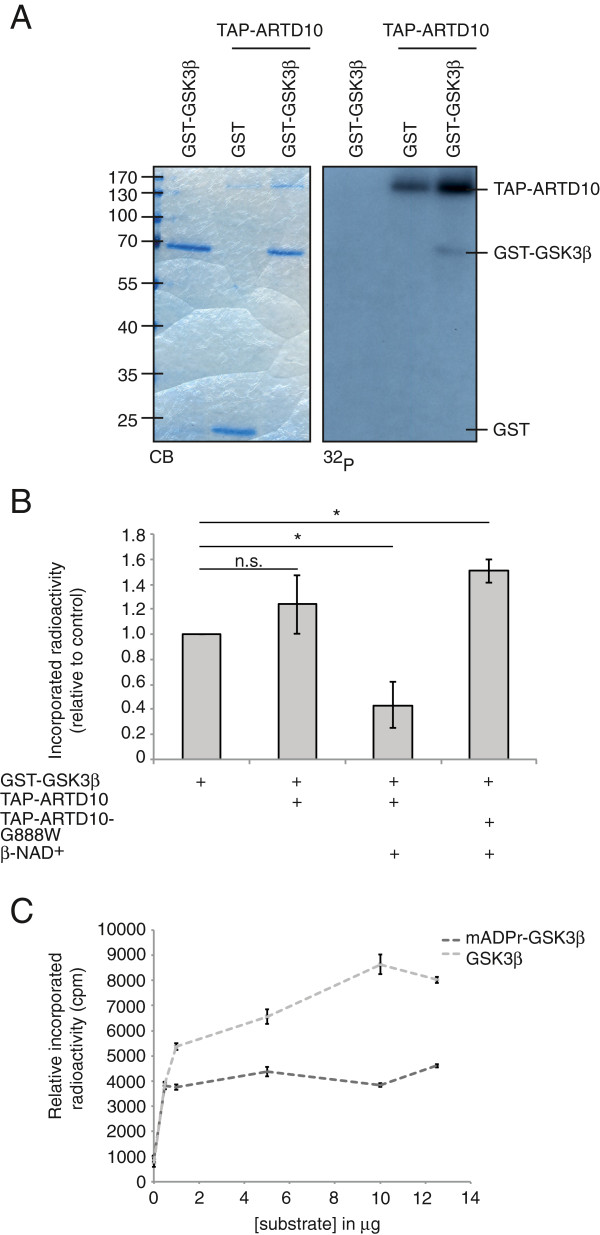
**ARTD10 mono-ADP-ribosylates GSK3β and thereby non-competitively inhibits its kinase activity *in vitro. *A.** ADP-ribosylation assay with [^32^P]-NAD^+^, TAP-ARTD10 and BSA or GST-GSK3β, analyzed by SDS-PAGE, coomassie blue (CB) staining and autoradiography. **B.** GST-GSK3β was mono-ADP-ribosylated and subsequently assessed in a kinase assay with a primed substrate peptide and [^32^P]-γ-ATP. Incorporated radioactivity was measured by scintillation counting. **C.** Substrate peptide was titrated as indicated in a kinase assay with untreated or mono-ADP-ribosylated GST-GSK3β and [^32^P]-γ-ATP. Incorporated radioactivity was measured by scintillation counting.

To investigate the consequence of mono-ADP-ribosylation, we focused on the modification of the ARTD10 substrate GSK3β. Because it is currently not possible to directly measure mono-ADP-ribosylation in cells by using tools like antibodies, we decided to focus on a kinase so that kinase activity can be determined in response to mono-ADP-ribosylation as an indirect mean to investigate the consequence of the modification. Furthermore, GSK3β is not only representative of the largest substrate group formed by kinases, but also phosphorylates the ARTD10-interacting oncoprotein MYC [33]. First, we tested whether recombinant GST-GSK3β purified from SF9 cells also serves as ARTD10 substrate in an *in vitro* ADP-ribosylation assay with TAP-ARTD10. GST-GSK3β but not GST was indeed substrate of ARTD10 (Figure 3A), whereas the purified GST-GSK3β incubated with ^32^P]-NAD in the absence of ARTD10 was not modified. Next we asked whether mono-ADP-ribosylation influences the enzymatic activity of GSK3β. GST-GSK3β was mono-ADP-ribosylated by TAP-ARTD10 and subsequently tested in a kinase assay with radioactively labeled ^32^P]-γ-ATP and a primed substrate peptide [27]. The kinase activity of mono-ADP-ribosylated GSK3β was reduced, in contrast to incubation with ARTD10 without β-NAD^+^ or with the catalytically inactive ARTD10-G888W, which resulted in a small increase in activity that might be due to increased kinase stability, caused by the physical presence of ARTD10 (Figure 3B). This demonstrated that mono-ADP-ribosylation can directly influence the enzymatic activity of a modified protein *in vitro*, which has not been shown for any substrate of intracellular mono-ADP-ribosyltransferases thus far. To understand how mono-ADP-ribosylation interfered with the catalytic activity of GSK3β, *in vitro* kinase assays were performed with increasing concentrations of substrate peptide. Even high amounts of peptide failed to restore kinase activity of mono-ADP-ribosylated GSK3β in these *in vitro* assays (Figure 3C), suggesting that mono-ADP-ribosylation is a non-competitive inhibitor. This is in contrast to Ser-9 phosphorylation of GSK3β, a site that is regulated by upstream signaling cascades and is a substrate of the AKT kinase, which functions as competitive inhibitor by occupying the site for the priming phosphate of the substrate [34,35]. This implies that mono-ADP-ribosylation potentially regulates GSK3β activity by a mechanism complementary to inhibition through Ser-9 phosphorylation.

### ARTD10 modulates GSK3β activity in cells

**Figure 4:**
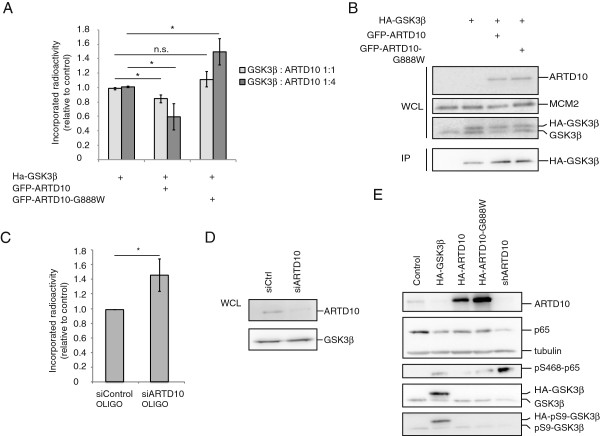
**Manipulation of ARTD10 levels reflects on GSK3β activity in cells. A.** GFP*-*ARTD10 or GFP-ARTD10-G888W was co-expressed with HA-GSK3β in U2OS cells. Immunoprecipitated HA-GSK3β was subsequently subjected to a kinase assay with a primed substrate peptide and [^32^P]-γ-ATP. Incorporated radioactivity was measured by scintillation counting. **B.** Western blot analysis of the ARTD10 and GSK3β expression and immunoprecipitation levels of the kinase tested in (A) **C.** U2OS cells were transfected with siARTD10 or siControl SMARTpools, GSK3β was immunoprecipitated and subjected to kinase assays with a primed substrate peptide and [^32^P]-γ-ATP. Incorporated radioactivity was measured by scintillation counting. **D.** Western blot analysis of the ARTD10 and GSK3β expression levels of the kinase tested in (C) **E.** U2OS cells were transfected with HA-ARTD10, HA-ARTD10-G888W, HA-GSK3β or ARTD10 shRNA. 48 hours after transfection cells were lysed and analyzed.

Our previous study revealed that ARTD10, but not ARTD10-G888W, interferes with cell proliferation [3], suggesting that mono-ADP-ribosylation by ARTD10 in cells is taking place and is vital for normal cell physiology. Therefore we co-expressed GFP-ARTD10 or GFP-ARTD10-G888W together with HA-GSK3β in U2OS cells and subsequently determined kinase activity of immunoprecipitated GSK3β. We measured lower kinase activity with co-expression of ARTD10 compared with controls (Figure 4A), indicating that ARTD10 modified GSK3β in cells and thereby inhibited kinase activity similar to the *in vitro* assays. In contrast, co-expression of high ARTD10-G888W amounts induced kinase activity, which might be due to a dominant-negative effect of the catalytically inactive ARTD10 (Figure 4A). GSK3β expression levels were not influenced by co-expression of different ARTD10 constructs and additionally, the immunoprecipitation efficiencies were also comparable (Figure 4B). To complement these overexpression experiments with tests on endogenous proteins, we performed an siRNA mediated knockdown of ARTD10. Reducing ARTD10 levels enhanced endogenous GSK3β activity (Figure 4C), without influencing GSKβ protein levels (Figure 4D), thereby also hinting at the existence of endogenous mono-ADP-ribosylation by ARTD10. To further validate these findings, we measured the phosphorylation of the NF-κB subunit RELA/p65 at Ser-468, a known GSK3β target [36]. ARTD10-G888W overexpression and knockdown of endogenous ARTD10 by shRNA (initial tests of pSUPER-constructs are displayed in Additional file 5: Figure S2) increased p65-Ser-468 phosphorylation (Figure 4E), supporting the concept that mono-ADP-ribosylation of GSK3β by ARTD10 inhibits kinase activity. Ser-468 phosphorylation of p65 negatively regulates basal NF-κB activity [36], implying that ARTD10 could influence basal NF-κB activity through regulation of GSK3β. Recent findings indicate that certain immune signals can induce ARTD10 expression [37,38], also suggesting that ARTD10 is involved in the immune response. It is currently not possible to directly demonstrate mono-ADP-ribosylation of GSK3β in cells due to lack of suitable reagents, but together these findings support the hypothesis that mono-ADP-ribosylation by ARTD10 inhibits GSK3β activity not only *in vitro* but also in cells. It remains to be assessed whether this reflects a general regulation of GSK3β or whether distinct subpopulations of GSK3β are affected, thus controlling specific pathways only without affecting others as has been described for regulation of GSK3β before [28].

## Discussion

Mono-ADP-ribosylation is a currently poorly characterized post-translational modification. It is not known which proteins serve as substrate of the mono-ARTDs in cells and what the consequences of mono-ADP-ribosylation are for these proteins. Furthermore suitable methods are lacking to investigate mono-ADP-ribosylation in cells. By employing a protein substrate screen based on ProtoArrays® presented here (Figure 1), we were able to define potential substrates for two mono-ARTDs, i.e. ARTD10 and ARTD8 (Figure 2 and Additional file 2: Table S1 and Additional file 3: Table S2). This method can be adapted for other members of the ARTD family and also for the ARTC protein family. This will allow the definition of a set of proteins as targets of individual ADP-ribosyltransferases and, by learning more about the substrate preferences of the different enzymes, their physiological roles can be unraveled.

The findings reported here are the first evidence that mono-ADP-ribosylation by mono-ARTDs can regulate the enzymatic activity of a substrate, both *in vitro* (Figure 3) and in cells (Figure 4). The inhibitory effect measured on GSK3β in cells was small but consistent in these assays, which could possibly be caused by enzymes that remove the mono-ADP-ribosylation from GSK3β. It is known that poly-ADP-ribose is degraded very rapidly in cells [2], however poly-ADP-ribose glycohydrolase (PARG) and ADP-ribosylhydrolase 3 (ARH3) display no activity towards mono-ADP-ribosylated proteins [39-41]. We expect that also mono-ADP-ribosylation is a reversible process and therefore postulate that enzymes exist that can remove this modification from a substrate. This may also occur during cell lysis, especially under the mild conditions applied here to preserve GSK3β kinase activity. However, because the nature of such enzyme(s) has not been described thus far, it is difficult to control their activity. Likewise it is also difficult to detect ubiquitination of proteins without overexpressing ubiquitin and inhibiting the deubiquitinating enzymes [42]. Alternatively, only a distinct sub-population of GSK3β might be mono-ADP-ribosylated and therefore the measured inhibition of kinase activity could be masked by the unmodified population. This aspect needs to be addressed once antibodies against mono-ADP-ribosylation become available. Future analysis has to reveal which GSK3β substrates are influenced most by its mono-ADP-ribosylation. Moreover it will be important to define to what extent the consequence of this modification overlaps with the inhibitory effect of Ser-9 phosphorylation as well as other regulatory mechanisms that target GSK3β [43-45].

Because such a remarkable number of kinases were identified as ARTD8 and ARTD10 substrate (Figure 1D), it is tempting to speculate that mono-ADP-ribosylation serves as a general regulator of kinase activity. This has to be tested, however, by assessing the effect of mono-ADP-ribosylation on these other identified kinases. Of interest is also the observation that 10 of the 32 substrates shared between ARTD10 and ARTD8 are kinases. We noticed that when these two transferases were co-expressed in cells, they co-localized through specific interaction of the macrodomains of ARTD8 with mono-ADP-ribosylated ARTD10 (Forst et al., Structure, in press). This might suggest that some of the substrates that we identified are co-regulated. It is also possible that these two transferases act successively. These aspects are in need of further analysis. The consequences of mono-ADP-ribosylation for the identified growth factors have also to be investigated in more detail, where it should also be addressed when ARTD10 is in spatial proximity to these potential substrates. Currently, it is not clear if and when ARTD10 and the identified growth factors interact to allow modification.

However, these findings imply that mono-ADP-ribosylation might have an important role to play in the regulation of multiple processes by modifying substrate proteins such as GSK3β, IKKε and PDGF-B. This makes the regulation of ARTD10 itself an interesting research question, because it is currently also not clear when ARTD10 is active. At the moment, this is difficult to study because no direct read-outs for ARTD10 activity in cells exist. Furthermore, mono-ADP-ribosylation cannot be measured as easily by mass spectrometry as for example phosphorylation [46,47], as also illustrated by the fact that publications that describe automodification sites in ARTD1 contradict each other [48,49]. To be able to fully understand the role of mono-ADP-ribosylation in cell physiology, reliable methods have to be developed to study this modification. With antibodies against mono-ADP-ribosylated amino acids, it could for example be investigated under which circumstances GSK3β is normally modified by ARTD10 in cells or whether the other *in vitro* substrates are indeed also ARTD10 targets in cells.

## Conclusions

In order to understand the PTM mono-ADP-ribosylation better, it is necessary to identify target proteins, which can be investigated with the screening method presented here. Furthermore, we establish mono-ADP-ribosylation as a PTM that can directly regulate the activity of a substrate kinase. This not only adds another dimension to the regulation of GSK3β, but also provides an example of the relevance of intracellular mono-ADP-ribosylation. Unfortunately, mono-ADP-ribosylation cannot be directly measured in cells at the moment. A major challenge for the field will thus be the development of antibodies recognizing mono-ADP-ribosylated residues, as well as of reliable methods for modification site mapping to enable the further analysis of endogenous mono-ADP-ribosylation by the ARTD family.

## Methods

### Cell lines and reagents

U2OS and HeLa cells were kept at a humidified atmosphere at 37°C with 5% CO_2_ at all times and were cultivated in DMEM supplemented with 10% heat-inactivated fetal calf serum and 1% penicillin/streptomycin. Transfections were performed using the calcium phosphate precipitation technique. shRNA mediated knockdown was achieved by co-transfection of two different pSUPER constructs targeting different regions [50]. Dharmacon siRNA pools were transfected using Lipofectamin (Invitrogen) according to manufacturer’s instructions at a final concentration of 50 nM.

### Cloning and mutagenesis

Cloning of ARTD10 constructs has been described before [3,24,51]. pSUPER-ARTD10_1 and pSUPER-ARTD10_6 were generated with the following oligonucleotides:

siARTD10_1_for: GAT CCC CGG GTA GAG GGA TTA TGA CAT TCA AGA GAT GTC ATA ATC CCT CTA CCC TTT TTG GAA A

siARTD10_1_rev: AGC TTT TCC AAA AAG GGT AGA GGG ATT ATG ACA TCT CTT GAA TGT CAT AAT CCC TCT ACC CGG G

siARTD10_2_for: GAT CCC CGT GCA GGG ACT GTG ACA ATT TCA AGA GAA TTG TCA CAG TCC CTG CAC TTT TTG GAA A

siARTD10_2_rev: AGC TTT TCC AAA AAG TGC AGG GAC TGT GAC AAT TCT CTT GAA ATT GTC ACA GTC CCT GCA CGG G

siARTD10_3_for: GAT CCC CCT TGA AGG ACC GGA TAT GAT TCA AGA GAT CAT ATC CGG TCC TTC AAG TTT TTG GAA A

siARTD10_3_rev: AGC TTT TCC AAA AAC TTG AAG GAC CGG ATA TGA TCT CTT GAA TCA TAT CCG GTC CTT CAA GGG G

siARTD10_4_for: GAT CCC CTG GGT CCC ATG GAG ATC ACT TCA AGA GAG TGA TCT CCA TGG GAC CCA TTT TTG GAA A

siARTD10_4_rev: AGC TTT TCC AAA AAT GGG TCC CAT GGA GAT CAC TCT CTT GAA GTG ATC TCC ATG GGA CCC AGG G

siARTD10_5_for: GAT CCC CAG TGG CAG AAC GAG TGT TGT TCA AGA GAC AAC ACT CGT TCT GCC ACT TTT TTG GAA A

siARTD10_5_rev: AGC TTT TCC AAA AAA GTG GCA GAA CGA GTG TTG TCT CTT GAA CAA CAC TCG TTC TGC CAC TGG G

siARTD10_6_for: GAT CCC CAG GCC TTG AAG AGG TGG ACT TCA AGA GAG TCC ACC TCT TCA AGG CCT TTT TTG GAA A

siARTD10_6_rev: AGC TTT TCC AAA AAA GGC CTT GAA GAG GTG GAC TCT CTT GAA GTC CAC CTC TTC AAG GCC TGG G

pcDNA3.1-HA-GSK3β Addgene plasmid 14753 [52] was used to create pDONR/zeo-GSK3β and pBAC-GST-GSK3β using the GateWay cloning system according to manufacturer’s recommendations. Primers used were GSK3_attB1 and GSK3_attB2.

GSK3_attB1: GGG GAC AAG TTT GTA CAA AAA AGC AGG CTT CAT GTC AGG GCG GCC CAG A

GSK3_attB2: GGG GAC CAC TTT GTA CAA GAA AGC TGG GTC TTA GGT GGA GTT GGA AGC TGA TGC AG

### Protein purification

Enzymatically active ARTD10 and the inactive ARTD10-G888W were purified using the TAP-tagging method as described before [3]. The GST-tagged catalytic domain or inactive mutant G888W, comprising amino acids 818-1025, were purified from *E.coli* strain Bl21, where protein expression was induced with 0.4 mM IPTG overnight at 20°C. GST-GSK3β was purified from insect SF9 cells infected with GST-GSK3β virus, produced after transfection of pBAC-GST-GSK3β by using BaculoGold (BD Biosciences) according to manufacturer’s instructions. Recombinant IKKε, GSK3 β and P-TEFb were purchased from Invitrogen. Recombinant His-ING5 was purified from *E.coli* strain Bl21, after induction of protein expression with 1 mM IPTG for 2 hours at 37°C. Recombinant ACVR1(171-499) was a gift from S. Knapp, recombinant PDGF-B was a gift from T. Ostendorf and recombinant His-ARTD8 was a gift from P.O. Hassa.

### Western blotting and antibodies

Cells were lysed in TAP-lysis buffer (50 mM Tris pH7.5, 150 mM NaCl, 1 mM EDTA, 10% (v/v) glycerol, 1% (v/v) NP-40, 1 mM DTT and 100 μM sodium vanadate) supplemented with ProteoBlock Protease inhibitor cocktail (Fermentas) and cleared by high-speed centrifugation. After boiling with SDS-sample buffer 10-18%-SDS polyacrylamide gel electrophoresis was used to separate the proteins followed by transfer to nitrocellulose membrane (Millipore). Blocking of the membrane was performed at RT for at least 30 minutes in 5% non-fat milk in TBS-T. Primary antibodies were incubated at 4°C overnight. Primary antibodies used are α-GSK3β (H-76, Santa Cruz), α-GSK3β-S9A (Cell Signaling), α-ARTD10 (5H11, monoclonal antibody raised in rat against ARTD10 fragment 2 (amino acids 206-459), α-tubulin (T6557, Sigma), α-p65 (3987, Cell Signaling), α-p65 phospho Ser 468 (3039, Cell Signaling), α-GFP (600-301-215, Rockland), α-HA (3 F10, Roche) and α-MCM2 (N-19, Santa Cruz).

### ADP-ribosylation assays

ADP-ribosylation assays were routinely carried out at 30°C for 30 minutes unless indicated otherwise. The reaction mixture (50 mM Tris-HCl, pH 8.0, 0.2 mM DTT, 5 mM MgCl_2_ and 50 μM β-NAD^+^ (Sigma) and 1 μCi [^32^P]-β-NAD^+^ (Amersham Biosciences)) was added to 0.5-1 μg purified substrate protein and 0.5 μg enzyme in a total reaction volume of 30 μl. Reactions were stopped by adding SDS sample buffer and were subsequently boiled and run on SDS-PAGE. Incorporated radioactivity was analyzed by exposure of the dried gel to X-ray film. Samples used in subsequent kinase assays were cooled on ice before washing of beads with coupled GST-GSK3β in kinase assay buffer (see below). Alternatively, 50 μM biotin-NAD^+^ (Trevigen) was added to the reaction mixture instead of regular β-NAD^+^ and [^32^P]-β-NAD^+^, followed by analysis by SDS-PAGE and Western Blot.

### Kinase assays

Kinase buffer: 5 mM MOPS pH 7.2, 2.5 mM β-glycerophosphate, 1 mM EGTA, 0.4 mM EDTA, 4 mM MgCl_2_, 50 μM DTT and 40 ng/μl BSA. [^32^P]-γ-ATP was diluted to 0.16 μCi/μl in 250 μM ATP in 3x kinase assay buffer. 25 ng GST-GSK3β or precipitated material was incubated in a reaction volume of 25 μl containing 5 μl 0.16 μCi/μl [^32^P]-γ-ATP-solution and 5 μg substrate peptide RRRPASVPPSPSLSRHS(pS)HQRR (Millipore), unless the substrate peptide was titrated in indicated concentrations. After incubating at 30°C for 15 minutes the reaction was stopped by placing on ice. Assays with immunoprecipitated material were spun-down before analysis to pellet the beads. Routinely 10 μl aliquots were spotted on P81 paper in duplicate, washed with 0.5% phosphoric acid and air-dried before scintillation counting. Beads with immunoprecipitated material were subsequently boiled and analyzed by Western Blot. Data of the experiments with recombinant proteins are represented as mean ± SD of at least triplicate measurements from a representative experiment. Data of experiments with immunoprecipitated material are represented as mean ± SEM of biological triplicates. Statistical significance was determined by employing two-sided Student’s t-tests (* = p < 0.05, n.s. = not significant).

### **ProtoArrays**®

ProtoArrays® (v4.1) were purchased from Invitrogen and contain approximately 8000 proteins spotted in duplicate on a thin layer of nitrocellulose. All solutions used were filtered through 0.22 μm filter devices prior to use to eliminate background-causing dust particles. Arrays were removed from -20°C and allowed to thaw at room temperature for 20 minutes. Roti-Block (Roth) was used to block the arrays by incubating for 1 hour at 4°C, shaking with 50 rpm. Arrays were washed once in plain reaction buffer (50 mM Tris-HCl, pH 8.0) and dried at the back and sides with Kim wipes. The reaction mixture (50 mM Tris-HCl, pH 8.0, 0.2 mM DTT, 4 mM MgCl_2_, 25 μM biotin-NAD^+^ and approximately 1.5 μg enzyme) was applied to the slides upon which LifterSlips (Nunc) were placed on the arrays to prevent evaporation of the reaction mixture. This was incubated for 1 hour at 30°C and subsequently washed 3x with 1% fatty-acid-free BSA in TBS/T, 3x with 0.5% SDS in TBS/T and again 3x with 1% fatty-acid-free BSA, each wash step taking 10 minutes. To detect the biotinylated proteins, streptavidin-AlexaFluor® 647 was applied in the dark for 90 minutes at 4°C at a concentration of 2 μg/ml in 1% fatty-acid-free BSA in TBS/T, after which five 10-minute wash steps with 1% fatty-acid-free BSA in TBST/T and 5 wash steps with ddH_2_O were performed. Arrays were centrifuged at 200xg for 2 minutes to remove residual ddH_2_O and subsequently analyzed using an Axon GenePix 4100A microarray reader with the GenePixPro 6.0 program. Results were analyzed with the Prospector software provided by Invitrogen.

Z-scores were calculated to assess statistical significance, according to the following formula: z = (x-μ)/σ where x is the raw value, μ the population mean and σ the standard deviation of the population. All proteins having a Z-score ≥ 2.5 were considered as a positive hit and were displayed in the Additional file 2: Table S1 and Additional file 3: Table S2. Lists containing the Uniprot/Swissprot IDs of all identified ARTD8 and ARTD10 substrates were generated and subsequently analyzed using the online Biocompendium high-throughput experimental data analysis platform (biocompendium.embl.de). Enriched gene ontology (GO) terms are displayed in the additional tables, with separate tables for ARTD8 and ARTD10 substrate sets and GO terms for biological processes or GO terms for molecular functions.

## Abbreviations

ACVR1: Activin receptor type-1; ART: ADP-ribosyltransferase; β-NAD^+^: Nicotinamide adenine dinucleotide; GSK3β: Glycogen synthase kinase β; IKK: IκB kinase; ING5: Inhibitor of growth protein 5; PAR: Poly-ADP-ribose; PDGF: Platelet-derived growth factor subunit B; P-TEFb: Positive transcription elongation factor b; TAP: Tandem-affinity purified.

## Competing interests

The authors declare no competing financial interests.

## Authors’ contributions

KLHF, HK, and AB established the screen; KLHF performed the GSK3β experiments; UL performed the ING5 experiment; AHF, NH, and PV purified ARTD10; EK generated the mAbs; BL supervised the work; KLHF and BL wrote the manuscript. All authors read and approved the final manuscript.

## Supplementary Material

Additional file 1 **Figure S1. **A. *In vitro* ADP-ribosylation assay performed with GST-ARTD10(818-1025) and biotin-NAD^+^, analyzed by SDS-PAGE an Western Blot. ADP-ribosylation was detected with HRP-coupled streptavidin, total protein with a GST-specific antibody. **B.** Three different sub-arrays are displayed for the arrays incubated with ARTD10 and the ARTD8. Spots of exemplary proteins indicated with green are enlarged. Click here for file

Additional file 2 **Table S1.** List of identified ARTD10 substrates. Click here for file

Additional file 3 **Table S2.** List of identified ARTD8 substrates. Click here for file

Additional file 4 **Table S3.** Summary of gene ontology analysis. Click here for file

Additional file 5 **Figure S2.** Six different shRNA constructs against ARTD10 were tested in HeLa cells on overexpressed HA-ARTD10. The ARTD10 protein levels were normalized against actin. Click here for file

## References

[B1] Hottiger MO Hassa PO Luscher B Schuler H Koch-Nolte F Toward a unified nomenclature for mammalian ADP-ribosyltransferases Trends Biochem Sci 2010 35 208 219 10.1016/j.tibs.2009.12.003 20106667

[B2] Schreiber V Dantzer F Ame JC de Murcia G Poly(ADP-ribose): novel functions for an old molecule Nat Rev Mol Cell Biol 2006 7 517 528 10.1038/nrm1963 16829982

[B3] Kleine H Poreba E Lesniewicz K Hassa PO Hottiger MO Litchfield DW Shilton BH Luscher B Substrate-assisted catalysis by PARP10 limits its activity to mono-ADP-ribosylation Mol Cell 2008 32 57 69 10.1016/j.molcel.2008.08.009 18851833

[B4] Kalisch T Ame JC Dantzer F Schreiber V New readers and interpretations of poly(ADP-ribosyl)ation Trends Biochem Sci 2012 37 381 390 10.1016/j.tibs.2012.06.001 22766145 PMC7127722

[B5] Hassa PO Haenni SS Elser M Hottiger MO Nuclear ADP-ribosylation reactions in mammalian cells: where are we today and where are we going? Microbiol Mol Biol Rev 2006 70 789 829 10.1128/MMBR.00040-05 16959969 PMC1594587

[B6] Gibson BA Kraus WL New insights into the molecular and cellular functions of poly(ADP-ribose) and PARPs Nat Rev Mol Cell Biol 2012 13 411 424 10.1038/nrm3376 22713970

[B7] Schreiber V Ame JC Dolle P Schultz I Rinaldi B Fraulob V Fraulob V Menissier-de Murcia J Poly(ADP-ribose) polymerase-2 (PARP-2) is required for efficient base excision DNA repair in association with PARP-1 and XRCC1 J Biol Chem 2002 277 23028 23036 10.1074/jbc.M202390200 11948190

[B8] Menissier de Murcia J Ricoul M Tartier L Niedergang C Huber A Dantzer F Schreiber V Ame JC Dierich A LeMeur M Functional interaction between PARP-1 and PARP-2 in chromosome stability and embryonic development in mouse EMBO J 2003 22 2255 2263 10.1093/emboj/cdg206 12727891 PMC156078

[B9] Huang SM Mishina YM Liu S Cheung A Stegmeier F Michaud GA Charlat O Wiellette E Zhang Y Wiessner S Tankyrase inhibition stabilizes axin and antagonizes Wnt signalling Nature 2009 461 614 620 10.1038/nature08356 19759537

[B10] Callow MG Tran H Phu L Lau T Lee J Sandoval WN Liu PS Bheddah S Tao J Lill JR Ubiquitin ligase RNF146 regulates tankyrase and Axin to promote Wnt signaling PLoS One 2011 6 e22595 10.1371/journal.pone.0022595 21799911 PMC3143158

[B11] Zhang Y Liu S Mickanin C Feng Y Charlat O Michaud GA Schirle M Shi X Hild M Bauer A RNF146 is a poly(ADP-ribose)-directed E3 ligase that regulates axin degradation and Wnt signalling Nat Cell Biol 2011 13 623 629 10.1038/ncb2222 21478859

[B12] Levaot N Voytyuk O Dimitriou I Sircoulomb F Chandrakumar A Deckert M Krzyzanowski PM Scotter A Gu S Janmohamed S Loss of Tankyrase-mediated destruction of 3BP2 is the underlying pathogenic mechanism of cherubism Cell 2011 147 1324 1339 10.1016/j.cell.2011.10.045 22153076 PMC3475183

[B13] Guettler S LaRose J Petsalaki E Gish G Scotter A Pawson T Rottapel R Sicheri F Structural basis and sequence rules for substrate recognition by Tankyrase explain the basis for cherubism disease Cell 2011 147 1340 1354 10.1016/j.cell.2011.10.046 22153077

[B14] Ferro AM Olivera BM Poly(ADP-ribosylation) in vitro Reaction parameters and enzyme mechanism J Biol Chem 1982 257 7808 7813 6282854

[B15] Aravind L The WWE domain: a common interaction module in protein ubiquitination and ADP ribosylation Trends Biochem Sci 2001 26 273 275 10.1016/S0968-0004(01)01787-X 11343911

[B16] Ahel I Ahel D Matsusaka T Clark AJ Pines J Boulton SJ West SC Poly(ADP-ribose)-binding zinc finger motifs in DNA repair/checkpoint proteins Nature 2008 451 81 85 10.1038/nature06420 18172500

[B17] Timinszky G Till S Hassa PO Hothorn M Kustatscher G Nijmeijer B Colombelli J Altmeyer M Stelzer EH Scheffzek K A macrodomain-containing histone rearranges chromatin upon sensing PARP1 activation Nat Struct Mol Biol 2009 16 923 929 10.1038/nsmb.1664 19680243

[B18] Ahel D Horejsi Z Wiechens N Polo SE Garcia-Wilson E Ahel I Flynn H Skehel M West SC Jackson SP Poly(ADP-ribose)-dependent regulation of DNA repair by the chromatin remodeling enzyme ALC1 Science 2009 325 1240 1243 10.1126/science.1177321 19661379 PMC3443743

[B19] Gottschalk AJ Timinszky G Kong SE Jin J Cai Y Swanson SK Washburn MP Florens L Ladurner AG Conaway JW Conaway RC Poly(ADP-ribosyl)ation directs recruitment and activation of an ATP-dependent chromatin remodeler Proc Natl Acad Sci USA 2009 106 13770 13774 10.1073/pnas.0906920106 19666485 PMC2722505

[B20] Kang HC Lee YI Shin JH Andrabi SA Chi Z Gagne JP Lee Y Ko HS Lee BD Poirier GG Iduna is a poly(ADP-ribose) (PAR)-dependent E3 ubiquitin ligase that regulates DNA damage Proc Natl Acad Sci USA 2011 108 14103 14108 10.1073/pnas.1108799108 21825151 PMC3161609

[B21] Wang Z Michaud GA Cheng Z Zhang Y Hinds TR Fan E Cong F Xu W Recognition of the iso-ADP-ribose moiety in poly(ADP-ribose) by WWE domains suggests a general mechanism for poly(ADP-ribosyl)ation-dependent ubiquitination Genes Dev 2012 26 235 240 10.1101/gad.182618.111 22267412 PMC3278890

[B22] Kleine H Luscher B Learning how to read ADP-ribosylation Cell 2009 139 17 19 10.1016/j.cell.2009.09.018 19804746

[B23] Welsby I Hutin D Leo O Complex roles of members of the ADP-ribosyl transferase super family in immune defences: looking beyond PARP1 Biochem Pharmacol 2012 84 11 20 10.1016/j.bcp.2012.02.016 22402301

[B24] Yu M Schreek S Cerni C Schamberger C Lesniewicz K Poreba E Vervoorts J Walsemann G Grotzinger J Kremmer E PARP-10, a novel Myc-interacting protein with poly(ADP-ribose) polymerase activity, inhibits transformation Oncogene 2005 24 1982 1993 10.1038/sj.onc.1208410 15674325

[B25] Kleine H Herrmann A Lamark T Forst AH Verheugd P Luscher-Firzlaff J Lippok B Feijs KL Herzog N Kremmer E Dynamic subcellular localization of the mono-ADP-ribosyltransferase ARTD10 and interaction with the ubiquitin receptor p62 Cell Commun Signal 2012 10 28 10.1186/1478-811X-10-28 22992334 PMC3508985

[B26] Chou HY Chou HT Lee SC CDK-dependent activation of poly(ADP-ribose) polymerase member 10 (PARP10) J Biol Chem 2006 281 15201 15207 10.1074/jbc.M506745200 16455663

[B27] Cohen P Frame S The renaissance of GSK3 Nat Rev Mol Cell Biol 2001 2 769 776 11584304 10.1038/35096075

[B28] Doble BW Woodgett JR GSK-3: tricks of the trade for a multi-tasking kinase J Cell Sci 2003 116 1175 1186 10.1242/jcs.00384 12615961 PMC3006448

[B29] Meng L Michaud GA Merkel JS Zhou F Huang J Mattoon DR Schweitzer B Protein kinase substrate identification on functional protein arrays BMC Biotechnol 2008 8 22 10.1186/1472-6750-8-22 18307815 PMC2270825

[B30] Saxty BA Yadollahi-Farsani M Upton PD Johnstone SR MacDermot J Inactivation of platelet-derived growth factor-BB following modification by ADP-ribosyltransferase Br J Pharmacol 2001 133 1219 1226 10.1038/sj.bjp.0704187 11498506 PMC1621139

[B31] Cho SH Goenka S Henttinen T Gudapati P Reinikainen A Eischen CM Lahesmaa R Boothby M PARP-14, a member of the B aggressive lymphoma family, transduces survival signals in primary B cells Blood 2009 113 2416 2425 10.1182/blood-2008-03-144121 19147789 PMC2656269

[B32] Cho SH Ahn AK Bhargava P Lee CH Eischen CM McGuinness O Boothby M Glycolytic rate and lymphomagenesis depend on PARP14, an ADP ribosyltransferase of the B aggressive lymphoma (BAL) family Proc Natl Acad Sci USA 2011 108 15972 15977 10.1073/pnas.1017082108 21911376 PMC3179111

[B33] Vervoorts J Luscher-Firzlaff J Luscher B The ins and outs of MYC regulation by posttranslational mechanisms J Biol Chem 2006 281 34725 34729 10.1074/jbc.R600017200 16987807

[B34] Dajani R Fraser E Roe SM Young N Good V Dale TC Pearl LH Crystal structure of glycogen synthase kinase 3 beta: structural basis for phosphate-primed substrate specificity and autoinhibition Cell 2001 105 721 732 10.1016/S0092-8674(01)00374-9 11440715

[B35] Frame S Cohen P Biondi RM A common phosphate binding site explains the unique substrate specificity of GSK3 and its inactivation by phosphorylation Mol Cell 2001 7 1321 1327 10.1016/S1097-2765(01)00253-2 11430833

[B36] Buss H Dorrie A Schmitz ML Frank R Livingstone M Resch K Kracht M Phosphorylation of serine 468 by GSK-3beta negatively regulates basal p65 NF-kappaB activity J Biol Chem 2004 279 49571 49574 10.1074/jbc.C400442200 15465828

[B37] Mahmoud L Al-Saif M Amer HM Sheikh M Almajhdi FN Khabar KS Green fluorescent protein reporter system with transcriptional sequence heterogeneity for monitoring the interferon response J Virol 2011 85 9268 9275 10.1128/JVI.00772-11 21752918 PMC3165742

[B38] Salazar JC Duhnam-Ems S La Vake C Cruz AR Moore MW Caimano MJ Velez-Climent L Shupe J Krueger W Radolf JD Activation of human monocytes by live Borrelia burgdorferi generates TLR2-dependent and -independent responses which include induction of IFN-beta PLoS Pathog 2009 5 e1000444 10.1371/journal.ppat.1000444 19461888 PMC2679197

[B39] Dunstan MS Barkauskaite E Lafite P Knezevic CE Brassington A Ahel M Hergenrother PJ Leys D Ahel I Structure and mechanism of a canonical poly(ADP-ribose) glycohydrolase Nat Commun 2012 3 878 22673905 10.1038/ncomms1889

[B40] Oka S Kato J Moss J Identification and characterization of a mammalian 39-kDa poly(ADP-ribose) glycohydrolase J Biol Chem 2006 281 705 713 16278211 10.1074/jbc.M510290200

[B41] Slade D Dunstan MS Barkauskaite E Weston R Lafite P Dixon N Ahel M Leys D Ahel I The structure and catalytic mechanism of a poly(ADP-ribose) glycohydrolase Nature 2011 477 616 620 10.1038/nature10404 21892188 PMC3184140

[B42] Ellison MJ Hochstrasser M Epitope-tagged ubiquitin A new probe for analyzing ubiquitin function J Biol Chem 1991 266 21150 21157 1718971

[B43] Beurel E Michalek SM Jope RS Innate and adaptive immune responses regulated by glycogen synthase kinase-3 (GSK3) Trends Immunol 2010 31 24 31 10.1016/j.it.2009.09.007 19836308 PMC2818223

[B44] Hur EM Zhou FQ GSK3 signalling in neural development Nat Rev Neurosci 2010 11 539 551 20648061 10.1038/nrn2870 PMC3533361

[B45] Wu D Pan W GSK3: a multifaceted kinase in Wnt signaling Trends Biochem Sci 2010 35 161 168 10.1016/j.tibs.2009.10.002 19884009 PMC2834833

[B46] Hengel SM Goodlett DR A Review of Tandem Mass Spectrometry Characterization of Adenosine Diphosphate-Ribosylated Peptides Int J Mass Spectrom 2012 312 114 121 22563295 10.1016/j.ijms.2011.06.003 PMC3341133

[B47] Rosenthal F Messner S Roschitzki B Gehrig P Nanni P Hottiger MO Identification of distinct amino acids as ADP-ribose acceptor sites by mass spectrometry Methods Mol Biol 2011 780 57 66 10.1007/978-1-61779-270-0_4 21870254

[B48] Altmeyer M Messner S Hassa PO Fey M Hottiger MO Molecular mechanism of poly(ADP-ribosyl)ation by PARP1 and identification of lysine residues as ADP-ribose acceptor sites Nucleic Acids Res 2009 37 3723 3738 10.1093/nar/gkp229 19372272 PMC2699514

[B49] Tao Z Gao P Liu HW Identification of the ADP-ribosylation sites in the PARP-1 automodification domain: analysis and implications J Am Chem Soc 2009 131 14258 14260 10.1021/ja906135d 19764761

[B50] Brummelkamp TR Bernards R Agami R A system for stable expression of short interfering RNAs in mammalian cells Science 2002 296 550 553 10.1126/science.1068999 11910072

[B51] Lesniewicz K Luscher-Firzlaff J Poreba E Fuchs P Walsemann G Wiche G Luscher B Overlap of the gene encoding the novel poly(ADP-ribose) polymerase Parp10 with the plectin 1 gene and common use of exon sequences Genomics 2005 86 38 46 10.1016/j.ygeno.2005.03.009 15953538

[B52] He X Saint-Jeannet JP Woodgett JR Varmus HE Dawid IB Glycogen synthase kinase-3 and dorsoventral patterning in Xenopus embryos Nature 1995 374 617 622 10.1038/374617a0 7715701

