# Supplementary figures and images for: ARTD10 substrate identification on protein microarrays: regulation of GSK3β by mono-ADP-ribosylation
Source: Cell Commun Signal. 2013 Jan 19;11:5. doi: 10.1186/1478-811X-11-5 (PMC3627616; doi:10.1186/1478-811X-11-5)

# Additonal figure 1

A

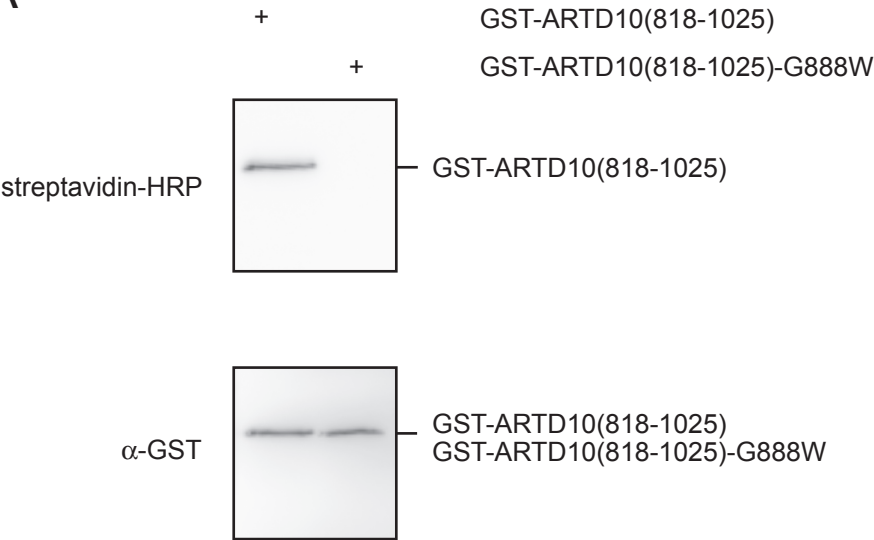

B

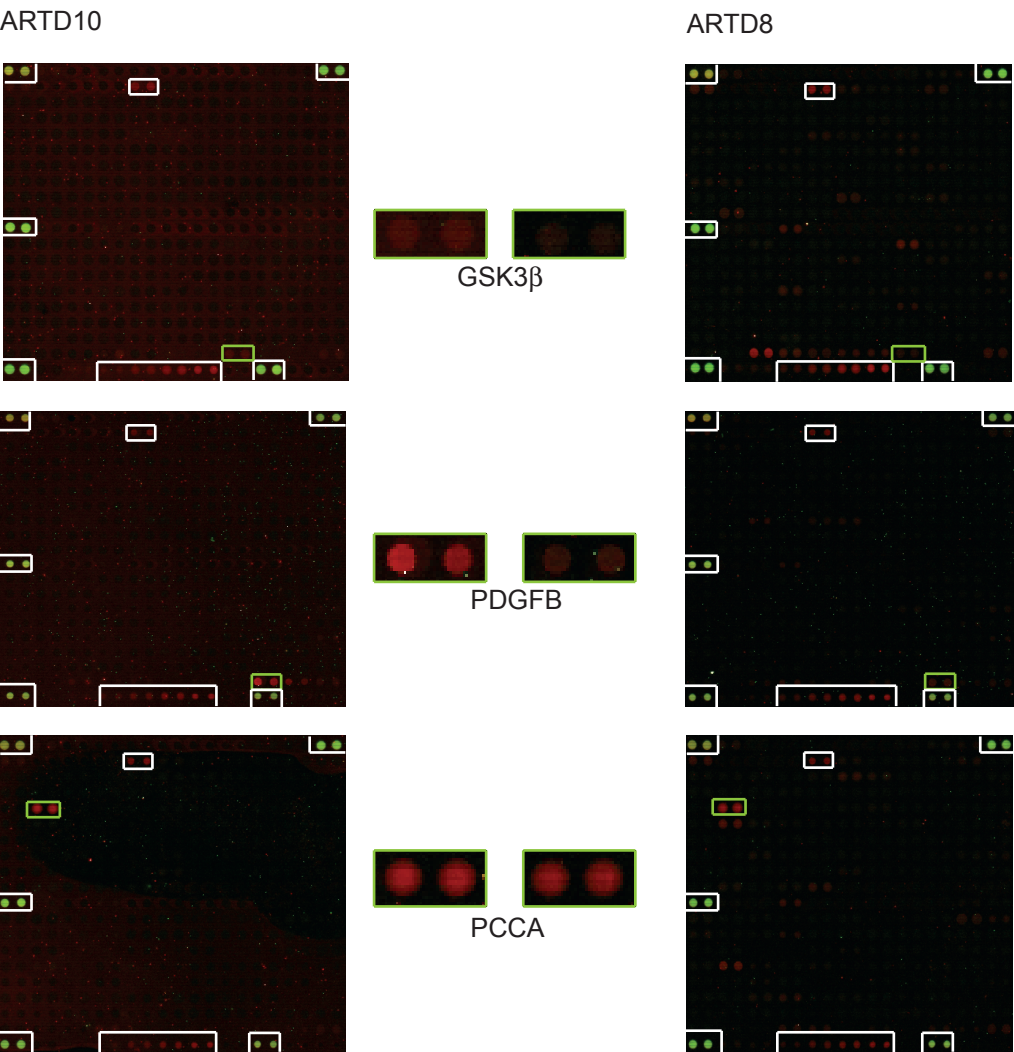

Supplement: Additional file 1 — Figure S1. A. In vitro ADP-ribosylation assay performed with GST-ARTD10(818-1025) and biotin-NAD+, analyzed by SDS-PAGE an Western Blot. ADP-ribosylation was detected with HRP-coupled streptavidin, total protein with a GST-specific antibody. B. Three different sub-arrays are displayed for the arrays incubated with ARTD10 and the ARTD8. Spots of exemplary proteins indicated with green are enlarged. [file 1478-811X-11-5-S1.pdf]

## Additional figure 2

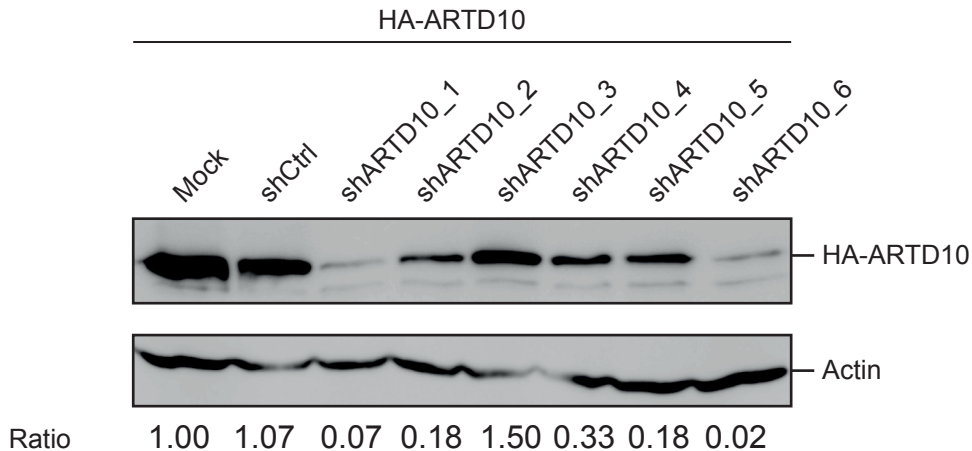

Supplement: Additional file 5 — Figure S2. Six different shRNA constructs against ARTD10 were tested in HeLa cells on overexpressed HA-ARTD10. The ARTD10 protein levels were normalized against actin. [file 1478-811X-11-5-S5.pdf]
