# Supplementary material for: ARTD10 substrate identification on protein microarrays: regulation of GSK3β by mono-ADP-ribosylation
Source: Cell Commun Signal. 2013 Jan 19;11:5. doi: 10.1186/1478-811X-11-5 (PMC3627616; doi:10.1186/1478-811X-11-5)
Supplement: Additional file 2 — Table S1. List of identified ARTD10 substrates. [file 1478-811X-11-5-S2.pdf]

Additional table 1: list of identified ARTD10 substrates

ProtoArray Identifie	Uniprot Identifier	Full protein name	Z-score	Comments
PHG0046	PDGFB_HUMAN	Platelet-derived growth factor subunit B	28,0	Validated
NM_000282.1	Q5JTW5_HUMAN	Propionyl coenzyme A carboxylase, alpha polypeptide	20,9	Biotin-binding enzyme
histone		Histone (unfractionated whole histone)	18,3	Known substrate
Histone_F2a2		Histones H2A and H4	9,8	Known substrate
PHC1244	CCL19_HUMAN	Chemokine (C-C motif) ligand 19	9,7	
PHC0215	IL21_HUMAN	Interleukin-21	9,4	
BC038838.1	PRR16_HUMAN	Proline-rich protein 16	7,5	
BC012109.1	HOME2_HUMAN	Homer protein homolog 2	7,0	
NM_020166.2	G5E9X5_HUMAN	Methylcrotonoyl-Coenzyme A carboxylase 1 (alpha) (MCCC1)	6,6	Biotin binding enzyme
NM_004113.3	FGF12_HUMAN	Fibroblast growth factor 12	6	
PV3612	STK6_HUMAN	Aurora kinase A	5,6	
PV3353	NEK6_HUMAN	Serine/threonine-protein kinase Nek6	5,5	
PV4877	ACVR1_HUMAN	Activin receptor type-1	5,3	Validated
PV3270	GSK3A_HUMAN	Glycogen synthase kinase alpha	5,1	
BC030711.2		C2orf13	5,0	
PV4883	ACVL1_HUMAN	Serine/threonine-protein kinase receptor R3	4,9	
PV3501	PLK1_HUMAN	Polo-like kinase 1	4,8	
NM_032459.1	EFS_HUMAN	Embryonal Fyn-associated substrate	4,8	
PV3973	KGP2_HUMAN	cGMP-dependent protein kinase 2	4,8	
NM_173597.1		Hypothetical protein FLJ37587 (FLJ37587)	4,8	
PV3878	MARK2_HUMAN	Serine/threonine-protein kinase MARK2	4,7	
PV3688	EPHA2_HUMAN	Ephrin type-A receptor 2	4,7	
PHC1346	SDF1_HUMAN	Stromal cell-derived factor 1 (CXCL12)	4,6	
PV3665	KC1D_HUMAN	Casein kinase I isoform delta	4,6	
P2287	KPCD_HUMAN	Protein kinase C delta type	4,6	
PHC1055	CCL5_HUMAN	C-C motif chemokine 5 (RANTES)	4,5	
PV3826	CLK3_HUMAN	Dual specificity protein kinase CLK3	4,4	
PHC0045	IL4_HUMAN	Interleukin-4	4,4	
BC025700.1	AFF4_HUMAN	AF/FMR2 family, member 4	4,2	
PV3365	GSK3B_HUMAN	Glycogen synthase kinase beta	4,2	Validated
PV4838	NR1I3_HUMAN	Nuclear receptor subfamily 1 group I member 3	4,2	
BC009327.2	NXN_HUMAN	Nucleoredoxin	4,1	
PV3371	BMX_HUMAN	Cytoplasmic tyrosine-protein kinase BMX	4,0	
NM_016096.1	ZN706_HUMAN	Zinc finger protein 706	4	
PV3857	KSYK_HUMAN	Tyrosine-protein kinase SYK	3,8	
PV3868	CDK7_HUMAN	Cyclin-dependent kinase 7	3,8	
PV3658	EPHB3_HUMAN	Ephrin type-B receptor 3	3,6	
La	LA_HUMAN	Lupus La protein	3,6	
PV3361	ARBK1_HUMAN	Beta-adrenergic receptor kinase 1	3,6	
PV4875	IKKE_HUMAN	Inhibitor of nuclear factor kappa-B kinase subunit epsilon	3,5	Validated
BC024725.1	ANR50_HUMAN	Ankyrin repeat domain-containing protein 5	3,5	
NM_004123.1	GIP_HUMAN	Gastric inhibitory polypeptide	3,5	
PV4762	THA_HUMAN	Thyroid hormone receptor alpha	3,5	
PHC1066		chemokine (C-X-C motif) ligand 1	3,5	
NM_005639.1	SYT1_HUMAN	Synaptotagmin-1	3,5	
PV4131	CCNT1_human	Cyclin T1	3,4	Validated
PV3686	DAPK3_HUMAN	Death-associated protein kinase 3	3,3	
PV3869	KIT_HUMAN	Mast/stem cell growth factor receptor Kit mutant protein: KIT T670I	3,3	
TopoI	TOP1_HUMAN	DNA topoisomerase 1	3,2	
PV3500	KC1E_HUMAN	Casein kinase I isoform epsilon	3,2	
PV3267	CDK2_HUMAN	Cyclin-dependent kinase 2	3,2	
P3081	KIT_HUMAN	Mast/stem cell growth factor receptor Kit	3,2	
BC020726.1	SCEL_HUMAN	Sciellin	3,2	
PV3144	NTRK1_HUMAN	High affinity nerve growth factor receptor	3,2	
BC008623.1	ROBO3_HUMAN	Roundabout homolog 3	3,2	
NM_152615.1	PAR15_HUMAN	Poly [ADP-ribose] polymerase 15	3,1	NAD-consuming enzyme
BC031691.2	SLAI2_HUMAN	SLAIN motif-containing protein 2	3,1	
XM_378350.2		hypothetical protein LOC400027 isoform 1	3,1	
PV3851	MARK4_HUMAN	MAP/microtubule affinity-regulating kinase 4	3,1	
PV3806	FER_HUMAN	Tyrosine-protein kinase Fer	3,1	
BC002448.2	ABLM1_HUMAN	Actin-binding LIM protein 1	3,1	
PV4205	KCC2B_HUMAN	Calcium/calmodulin-dependent protein kinase type II subunit beta	3,1	
NM_172160.1	KCAB1_HUMAN	Voltage-gated potassium channel subunit beta-1	3,1	
NM_004329.1	BMR1A_HUMAN	Bone morphogenetic protein receptor type-1A	3,1	
NM_032765.1	TRI52_HUMAN	Tripartite motif-containing protein 52	3,1	
BC062353.1		C1orf131	3,1	
PV4823	MELK_HUMAN	Maternal embryonic leucine zipper kinase	3,0	
PV3969	DAPK1_human	Death-associated protein kinase 1	3,0	
PV3146	FGFR1_HUMAN	Fibroblast growth factor receptor 1	3,0	
NM_144602.1		C16orf78	3	

NM_017588.1	WDR5_HUMAN	WD repeat-containing protein 5	3	
NM_002013.2	FKB15_HUMAN	FK506-binding protein 15	2,9	
NM_003403.3	TYY1_HUMAN	Transcriptional repressor protein YY1	2,8	
PV3000	CDK5_human	Cyclin-dependent kinase 5	2,8	
BC005008.1	CEAM5_HUMAN	Carcinoembryonic antigen-related cell adhesion molecule 5	2,7	
NM_206834.1		C6orf201	2,7	
BC000931.2	ATPG_HUMAN	ATP synthase subunit gamma, mitochondrial	2,7	
PV4788	C9JAM9_HUMAN	Phosphoinositide-3-kinase, catalytic, alpha polypeptide	2,6	
BC004925.1	GPC5C_HUMAN	G-protein coupled receptor family C group 5 member C	2,6	
P2291	KPCB_HUMAN	Protein kinase C beta type	2,6	
NM_138818.1	PRUNE_HUMAN	Protein prune homolog	2,5	
