# Supplementary material for: ARTD10 substrate identification on protein microarrays: regulation of GSK3β by mono-ADP-ribosylation
Source: Cell Commun Signal. 2013 Jan 19;11:5. doi: 10.1186/1478-811X-11-5 (PMC3627616; doi:10.1186/1478-811X-11-5)
Supplement: Additional file 3 — Table S2. List of identified ARTD8 substrates. [file 1478-811X-11-5-S3.pdf]

Additional table 2: list of identified ARTD8 substrates

ProtoArray Identifier	Uniprot Identifier	Full-length protein name	Z-score	Comments
CENPB	CENPB_HUMAN	Major centromere autoantigen B	26,1	
NM_138565.1	SRC8_HUMAN	Src substrate cortactin	21,6	
NM_000282.1	Q5JTW5_HUMAN	Propionyl coenzyme A carboxylase, alpha polypeptide	21,3	Biotin-binding enzyme
NM_032345.1	F8VT68_HUMAN	Within bgcn homolog (Drosophila)	15,0	
NM_007285.5	GBRL2_HUMAN	Gamma-aminobutyric acid receptor-associated protein-like 2	13,9	
NM_003403.3	TYY1_HUMAN	Transcriptional repressor protein YY1	11,6	
NM_004965.3	HMGN1_HUMAN	Non-histone chromosomal protein HMG-14	10,8	
NM_152434.1	F8W6W6_HUMAN	CWF19-like 2, cell cycle control (S. pombe)	9,9	
NM_015640.1	PAIRB_HUMAN	Plasminogen activator inhibitor 1 RNA-binding protein	9,3	
Histone_F2a2		Histones H2A and H4	9,2	
NM_032141.1	NSRP1_HUMAN	Nuclear speckle splicing regulatory protein 1	8,9	
NM_144659.1	TCP1L_HUMAN	T-complex protein 10A homolog 2	8,6	
BC005004.1	FA64A_HUMAN	Protein FAM64A	8,6	
NM_133336.1	NSD2_HUMAN	Probable histone-lysine N-methyltransferase NSD2	8,5	
PV4552	VDR_HUMAN	Vitamin D3 receptor	8,4	
histone		Histone (unfractionated whole histone)	8,2	
BC030711.2		C2orf13	8,1	
PV3186	KPCI_HUMAN	Protein kinase C iota type	8,0	
PV4762	THA_HUMAN	Thyroid hormone receptor alpha	7,8	
NM_024692.3	CLIP4_HUMAN	CAP-Gly domain-containing linker protein 4	7,8	
BC008623.1	ROBO3_HUMAN	Roundabout homolog 3	7,7	
P3049	ABL1_HUMAN	Tyrosine-protein kinase ABL1	7,7	
NM_002013.2	FKB15_HUMAN	FK506-binding protein 15	7,2	
NM_004113.3	FGF12_HUMAN	Fibroblast growth factor 12	7,2	
NM_032350.3		C7orf50	7,0	
NM_005639.1	SYT1_HUMAN	Synaptotagmin-1	6,8	
NM_145865.1	ANS4B_HUMAN	Ankyrin repeat and SAM domain-containing protein 4B	6,7	
NM_015933.1	CCD72_HUMAN	Coiled-coil domain-containing protein 72	6,6	
BC053895.1	IRS1_HUMAN	Insulin receptor substrate 1	6,5	
PV3973	KGP2_HUMAN	cGMP-dependent protein kinase 2	6,4	
PV3366	F5H1T4_HUMAN	V-erb-b2 erythroblastic leukemia viral oncogene homolog 2	6,3	
NM_003295.1	TCTP_HUMAN	Translationally-controlled tumor protein	6,3	
NM_017949.1	CUED1_HUMAN	CUE domain-containing protein 1	6,3	
NM_001023.2	E5RIP1_HUMAN	Ribosomal protein S20	6,2	
BC020221.1	STAC_HUMAN	SH3 and cysteine-rich domain-containing protein	5,7	
BC010125.1		C3orf37	5,6	
NM_014583.2	LMCD1_HUMAN	LIM and cysteine-rich domains protein 1	5,5	
PV3249	CSF1R_HUMAN	Macrophage colony-stimulating factor 1 receptor	5,5	
TopoI	TOP1_HUMAN	DNA topoisomerase 1	5,2	
PV3967	FLT3_HUMAN	Receptor-type tyrosine-protein kinase FLT3; D835Y	5,2	
BC051688.1		hypothetical protein FLJ10781	5,2	
BC033758.1	Q2V6Q1_HUMAN	Centaurin alpha 2	5,1	
PV4211	M4K2_HUMAN	Mitogen-activated protein kinase kinase kinase kinase 2	5,1	
BC025700.1	AFF4_HUMAN	AF/FMR2 family, member 4	5,1	
BC017070.1	CLUA1_HUMAN	Clusterin-associated protein 1	5,0	
PV4131	CCNT1_human	Cyclin T1	5,0	
NM_080390.3	TCAL2_HUMAN	Transcription elongation factor A protein-like 2	5,0	
NM_134323.1	TRBP2_HUMAN	RISC-loading complex subunit TARBP2	4,9	
PV3815	B4DLT4_HUMAN	Ribosomal protein S6 kinase, 70kDa, polypeptide 1	4,9	
BC024725.1	ANR50_HUMAN	Ankyrin repeat domain-containing protein 5	4,8	
NM_020166.2	G5E9X5_HUMAN	Methylcrotonoyl-Coenzyme A carboxylase 1 (alpha) (MCCC1)	4,8	Biotin-binding enzyme
NM_178151.1	DCX_HUMAN	Neuronal migration protein doublecortin	4,7	
PV3869	KIT_HUMAN	Mast/stem cell growth factor receptor Kit mutant protein: KIT T670I	4,7	
NM_017588.1	WDR5_HUMAN	WD repeat-containing protein 5	4,7	
PHG0046	PDGFB_HUMAN	Platelet-derived growth factor subunit B	4,6	
PV3879	PKN2_HUMAN	Serine/threonine-protein kinase N2	4,5	
NM_002822.1	TWF1_HUMAN	Twinfilin-1	4,5	
PV3299	MAPK3_HUMAN	MAP kinase-activated protein kinase 3	4,5	
PV3792	TTK_HUMAN	Dual specificity protein kinase TTK	4,5	
NM_018553.1		C17orf85	4,5	
NM_015414.2	(RL36_HUMAN)	60S ribosomal protein L36	4,5	
PV3836	IKKB_HUMAN	Inhibitor of nuclear factor kappa-B kinase subunit beta	4,5	
PV3822	M3K2_HUMAN	Mitogen-activated protein kinase kinase kinase 2	4,4	
PV4792	SIK2_HUMAN	Serine/threonine-protein kinase SIK2	4,4	
PV3829	SRPK2_HUMAN	SFRS protein kinase 2 (SRPK2), transcript variant 2	4,4	
NM_005801.2	EIF1_HUMAN	Eukaryotic translation initiation factor 1	4,4	
PV4130	TOPK_HUMAN	Lymphokine-activated killer T-cell-originated protein kinase	4,4	
P2251	KPCB_HUMAN	Protein kinase C beta type	4,3	
NM_021627.2	SENP2_HUMAN	Sentrin-specific protease 2	4,3	
BC026345.1	ERMIN_HUMAN	Ermin	4,3	
PV3857	KSYK_HUMAN	Tyrosine-protein kinase SYK	4,3	
BC001728.1	TFPT_HUMAN	TCF3 fusion partner	4,3	
NM_032765.1	TRI52_HUMAN	Tripartite motif-containing protein 52	4,3	
PV4129	VGFR3_HUMAN	Vascular endothelial growth factor receptor 3	4,2	
NM_199124.1		C11orf63	4,2	
BC056415.1	RPAP3_HUMAN	RNA polymerase II-associated protein 3	4,2	

PV3371	BMX_HUMAN	Cytoplasmic tyrosine-protein kinase BMX	4,1	
BC017227.1	PHLP_HUMAN	Phosducin-like protein	4,1	
PV3808	INSRR_HUMAN	Insulin receptor-related protein	4,1	
NM_020836.2	BEGIN_HUMAN	Brain-enriched guanylate kinase-associated protein	4,1	
NM_013242.1		C16orf80	4,1	
NM_025241.1	UBXN6_HUMAN	UBX domain-containing protein 6	4,1	
PV4883	ACVL1_HUMAN	Serine/threonine-protein kinase receptor R3	4,1	
BC001132.1	DDX54_HUMAN	ATP-dependent RNA helicase DDX54	4,0	
BC050616.1	TSSC4_HUMAN	Protein TSSC4	4,0	
PV3817	WEE1_HUMAN	Wee1-like protein kinase	4,0	
BC062353.1		C1orf131	4,0	
NM_005038.1	PPID_HUMAN	Peptidyl-prolyl cis-trans isomerase D	3,9	
PV3660	VGFR2_HUMAN	Vascular endothelial growth factor receptor 2	3,9	
PV3146	FGFR1_HUMAN	Fibroblast growth factor receptor 1	3,8	
NM_001571.2	IRF3_HUMAN	Interferon regulatory factor 3	3,8	
PV3657	STK25_HUMAN	Serine/threonine-protein kinase 25	3,8	
NM_014047.1		C19orf53	3,6	
NM_198501.1	SMTL2_HUMAN	Smoothelin-like protein 2	3,6	
PV4674	AAPK2_HUMAN	5'-AMP-activated protein kinase catalytic subunit alpha-2	3,6	
NM_016096.1	ZN706_HUMAN	Zinc finger protein 706	3,6	
P2287	KPCD_HUMAN	Protein kinase C delta type	3,6	
BC000479.1	B7Z5R1_HUMAN	V-akt murine thymoma viral oncogene homolog 1	3,6	
PV3785	DYR1A_HUMAN	Dual specificity tyrosine-phosphorylation-regulated kinase 1A	3,6	
PV3501	PLK1_HUMAN	Polo-like kinase 1	3,5	
BC001396.1		C9orf32	3,5	
PV4310	IKKA_HUMAN	Inhibitor of nuclear factor kappa-B kinase subunit alpha	3,5	
P2268	KPCZ_HUMAN	Protein kinase C zeta type	3,5	
PV3251	EPHB4_HUMAN	Ephrin type-B receptor 4	3,5	
PV3878	MARK2_HUMAN	Serine/threonine-protein kinase MARK2	3,5	
NM_001014.2	RS10_HUMAN	40S ribosomal protein S10	3,5	
BC009762.2	TRI41_HUMAN	E3 ubiquitin-protein ligase TRIM41	3,4	
NM_003616.2	G5EA29_HUMAN)	Survival of motor neuron protein interacting protein 1	3,4	
NM_016287.2	HP1B3_HUMAN	Heterochromatin protein 1-binding protein 3	3,4	
NM_003992.1	CLK3_HUMAN	Dual specificity protein kinase CLK3	3,4	
PV4297	DCLK2_HUMAN	Serine/threonine-protein kinase DCLK2	3,4	
NM_016483.3	PHF7_HUMAN	PHD finger protein 7	3,4	
NM_018990.1		CXorf9	3,4	
PV3789	PAK3_HUMAN	Serine/threonine-protein kinase PAK 3	3,4	
NM_024946.1	F192A_HUMAN	Protein FAM192A	3,4	
PV3292	CDK1_HUMAN	Cyclin-dependent kinase 1	3,4	
NM_152615.1	PAR15_HUMAN	Poly [ADP-ribose] polymerase 15	3,4	NAD-consuming enzyme
PV4803	EGFR_HUMAN	Epidermal growth factor receptor	3,4	
P2282	KPCE_HUMAN	Protein kinase C epsilon type	3,3	
BC060828.1	ARI3A_HUMAN	AT-rich interactive domain-containing protein 3A	3,3	
NM_144594.1	GTSF1_HUMAN	Gametocyte-specific factor 1	3,3	
BC064984.1	ASXL1_HUMAN	Putative Polycomb group protein ASXL1	3,3	
PV3819	RET_HUMAN	Proto-oncogene tyrosine-protein kinase receptor Ret	3,3	
BC011707.1	NRBF2_HUMAN	Nuclear receptor-binding factor 2	3,2	
P2782	ZAP70_HUMAN	Tyrosine-protein kinase ZAP-70	3,2	
BC029046.1	H10_HUMAN	Histone H1.0	3,2	
PV3266	ABL2_HUMAN	Abelson tyrosine-protein kinase 2	3,2	
PV3354	FES_HUMAN	Tyrosine-protein kinase Fes/Fps	3,2	
BC020726.1	SCEL_HUMAN	Sciellin	3,2	
PV3851	MARK4_HUMAN	MAP/microtubule affinity-regulating kinase 4	3,2	
PV4680	PGFRA_HUMAN	Platelet-derived growth factor receptor alpha	3,2	
NM_032329.4	ING5_HUMAN	Inhibitor of growth protein 5	3,2	Validated
BC011842.2		hypothetical protein FLJ11184	3,1	
BC031691.2	SLAI2_HUMAN	SLAIN motif-containing protein 2	3,1	
NM_007045.2	FR1OP_HUMAN	FGFR1 oncogene partner	3,1	
NM_033414.1	ZN622_HUMAN	Zinc finger protein 622	3,1	
NM_152763.2		C1orf62	3,1	
NM_002363.1	MAGB1_HUMAN	Melanoma-associated antigen B1	3,1	
BC009967.1	NTKL_HUMAN	N-terminal kinase-like protein	3,1	
PV3301	MAPK5_HUMAN	MAP kinase-activated protein kinase 5	3,0	
PV3811	PGFRA_HUMAN	Platelet-derived growth factor receptor alpha	3,0	
BC011414.1		C5orf3	3,0	
NM_014481.2	APEX2_HUMAN	DNA-(apurinic or apyrimidinic site) lyase 2	3,0	
BC051868.2	UBE2O_HUMAN	Ubiquitin-conjugating enzyme E2 O	3,0	
NM_023015.3	INT3_HUMAN	Integrator complex subunit 3	3,0	
