# Supplementary material for: ARTD10 substrate identification on protein microarrays: regulation of GSK3β by mono-ADP-ribosylation
Source: Cell Commun Signal. 2013 Jan 19;11:5. doi: 10.1186/1478-811X-11-5 (PMC3627616; doi:10.1186/1478-811X-11-5)
Supplement: Additional file 4 — Table S3. Summary of gene ontology analysis. [file 1478-811X-11-5-S4.pdf]

Additional Table 3A: ARTD10 substrates - biological process

Accession	Term	Adjusted P-Value
GO:0007167	enzyme linked receptor protein signaling pathway	6e-05
GO:0032943	mononuclear cell proliferation	9e-04
GO:0046777	protein amino acid autophosphorylation	3e-03
GO:0007242	intracellular signaling cascade	5e-03
GO:0007243	protein kinase cascade	5e-03
GO:0007169	transmembrane receptor protein tyrosine kinase signaling pathway	5e-03
GO:0030509	BMP signaling pathway	1e-02
GO:0010463	mesenchymal cell proliferation	1e-02
GO:0007179	transforming growth factor beta receptor signaling pathway	1e-02
GO:0007166	cell surface receptor linked signal transduction	1e-02
GO:0050730	regulation of peptidyl-tyrosine phosphorylation	1e-02
GO:0016055	Wnt receptor signaling pathway	2e-02
GO:0030182	neuron differentiation	3e-02
GO:0002521	leukocyte differentiation	3e-02
GO:0000278	mitotic cell cycle	4e-02

Additional Table 3B: ARTD10 substrates - molecular function

Accession	Term	Adjusted P-Value
GO:0004713	protein tyrosine kinase activity	3e-28
GO:0004674	protein serine/threonine kinase activity	2e-27
GO:0016772	transferase activity, transferring phosphorus-containing groups	4e-20
GO:0032553	ribonucleotide binding	2e-13
GO:0017076	purine nucleotide binding	5e-13
GO:0050321	tau-protein kinase activity	9e-06
GO:0005057	receptor signaling protein activity	7e-05
GO:0046332	SMAD binding	1e-03
GO:0019838	growth factor binding	1e-03
GO:0005102	receptor binding	3e-03
GO:0017002	activin receptor activity	5e-03
GO:0004715	non-membrane spanning protein tyrosine kinase activity	9e-03
GO:0004697	protein kinase C activity	2e-02
GO:0005021	vascular endothelial growth factor receptor activity	4e-02

GO:0005003	ephrin receptor activity	4e-02
GO:0004887	thyroid hormone receptor activity	5e-02
GO:0005136	interleukin-4 receptor binding	6e-02
GO:0035173	histone kinase activity	6e-02
GO:0004696	glycogen synthase kinase 3 activity	6e-02
GO:0005020	stem cell factor receptor activity	6e-02
GO:0004693	cyclin-dependent protein kinase activity	7e-02
GO:0005134	interleukin-2 receptor binding	9e-02
GO:0003708	retinoic acid receptor activity	9e-02
GO:0042802	identical protein binding	9e-02
GO:0048184	follistatin binding	9e-02
GO:0047696	beta-adrenergic receptor kinase activity	9e-02

Additional Table 3C: ARTD8 substrates - biological process

**Accession**	**Term**	**Adjusted P-Value**
GO:0006468	protein amino acid phosphorylation	3e-33
GO:0043687	post-translational protein modification	3e-25
GO:0007169	transmembrane receptor protein tyrosine kinase signaling pathway	1e-07
GO:0007167	enzyme linked receptor protein signaling pathway	2e-07
GO:0018193	peptidyl-amino acid modification	1e-06
GO:0008152	metabolic process	5e-04
GO:0007242	intracellular signaling cascade	5e-04
GO:0007243	protein kinase cascade	6e-04
GO:0046777	protein amino acid autophosphorylation	9e-04
GO:0065007	biological regulation	1e-03
GO:0032502	developmental process	4e-03
GO:0050730	regulation of peptidyl-tyrosine phosphorylation	4e-03
GO:0048008	platelet-derived growth factor receptor signaling pathway	4e-02
GO:0010463	mesenchymal cell proliferation	4e-02
GO:0007186	G-protein coupled receptor protein signaling pathway	6e-02
GO:0040011	locomotion	8e-02

Additional Table 3D: ARTD8 substrates - molecular function

**Accession**	**Term**	**Adjusted P-Value**
GO:0004713	protein tyrosine kinase activity	1e-38
GO:0004674	protein serine/threonine kinase activity	4e-38
GO:0016772	transferase activity, transferring	2e-26

	phosphorus-containing groups	
GO:0032553	ribonucleotide binding	3e-16
GO:0017076	purine nucleotide binding	2e-15
GO:0019199	transmembrane receptor protein kinase activity	2e-09
GO:0004715	non-membrane spanning protein tyrosine kinase activity	3e-09
GO:0005515	protein binding	1e-06
GO:0005021	vascular endothelial growth factor receptor activity	8e-06
GO:0004697	protein kinase C activity	2e-05
GO:0008384	IkappaB kinase activity	3e-03
GO:0000287	magnesium ion binding	7e-03
GO:0005488	binding	9e-03
GO:0042802	identical protein binding	1e-02
GO:0005057	receptor signaling protein activity	1e-02
GO:0003824	catalytic activity	1e-02
GO:0005161	platelet-derived growth factor receptor binding	3e-02
GO:0046982	protein heterodimerization activity	6e-02
GO:0019838	growth factor binding	6e-02
GO:0004712	protein serine/threonine/tyrosine kinase activity	6e-02
GO:0004709	MAP kinase kinase kinase activity	8e-02
GO:0008434	vitamin D3 receptor activity	8e-02
GO:0046983	protein dimerization activity	8e-02
GO:0004710	MAP/ERK kinase kinase activity	8e-02
GO:0005020	stem cell factor receptor activity	8e-02
GO:0004699	calcium-independent protein kinase C activity	8e-02
